# Dense fine speckled nuclear immunofluorescence: A mildly reassuring antinuclear antibody pattern meriting consideration

**DOI:** 10.1002/iid3.1026

**Published:** 2023-09-26

**Authors:** Aleksandra Fijałkowska, Robert A. Schwartz, Anna Woźniacka

**Affiliations:** ^1^ Department of Dermatology and Venereology Medical University of Łódź Lodz Poland; ^2^ Department of Dermatology Rutgers New Jersey Medical School Newark New Jersey USA

**Keywords:** anti‐DFS70 antibody assay, antinuclear antibodies, connective tissue diseases, Sjögren's syndrome, systemic lupus erythematosus, systemic sclerosis

## Abstract

**Introduction:**

Antinuclear antibodies (ANAs) are regarded as a hallmark of connective tissue diseases (CTDs) and play a key role in their diagnosis, but the value of some particular antibodies in management of patients and the disease prognosis is controversial. The mechanism underlying the production of ANAs in CTDs, other chronic inflammatory conditions and even in healthy people, is not completely elucidated. Anti‐DFS70 antibodies connected with the dense fine speckled autoantigen of 70 kD, known as the lens epithelium‐derived growth factor p75, are a subgroup of ANAs. Their presence and coexistence with other antibodies and their clinical significance are the matter of debate.

**Methods:**

Based on literature data, the authors focused on current knowledge explaining the role of anti‐DFS70 antibodies in selected CTDs.

**Results:**

However, the literature data is ambiguous and does not fully support the validity of the anti‐DFS70 assay for a specific CTD diagnosis. Most researchers claim that the presence of anti‐DFS70 as the only one usually exclude the diagnosis of CTD. Nevertheless, its coexistence with other ANAs is not an excluding factor but has predictive value due to more favorable course of CTD. Such situations may also suggest an enhanced risk of the development of a CTD in the future.

**Conclusions:**

Although more studies are needed in this field, it seems reasonable to ascertain the presence of anti‐DFS70 in routine clinical practice.

## Graphical abstract

The hallmark of autoimmune connective tissue diseases is the production of many different circulating antinuclear antibodies. A newly emphasized antinuclear antibody with its dense fine speckled nuclear immunofluorescence pattern may constitute a differentiating factor, with an anti‐DFS70 antibody assay distinguishing healthy people and autoimmune connective tissue diseases patients among individuals who are ANA‐positive

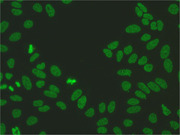

## 1 INTRODUCTION

Connective tissue diseases (CTD) are a group of heterogenous clinical disorders, which hallmark is the production of a baffling array of different circulating antinuclear autoantibodies (ANAs).^1^ An  ANA that has come to the forefront sparking considerable interest is the antibody with monospecific dense fine speckled (DFS) pattern visible in indirect immunofluorescence (IIF), as it has been identified as a possible indicator that an individual has a low probability of a concerning antinuclear antibody ‐associated rheumatologic disease.^2^ This DFS pattern is linked with autoantibodies against transcription co‐activator DFS70/Lens‐epithelial derived growth factor (LEDGF)p75.

However, there is considerable interassay variability for these autoantibodies in clinical laboratories.^3^

Despite their historical name, ANAs are directed against a wide spectrum of intracellular antigens, which include both the structures of the cell nucleus and the cytoplasm.^4^ The initial identification of ANAs by IIF remains the gold standard in the immunodiagnosis of CTDs. ANAs are important not only in the diagnosis but also often in prognosis.^1^ Accordingly, current research is focused on establishing connections between the clinical picture of the disease and the presence of specific ANAs in a patient's serum.^4^, ^5^, ^6^ Some antibodies exhibit high specificity and, therefore, have high diagnostic value.^7^ Most of them, however, are not specific to any individual disease.^8^ Moreover, ANAs are sometimes found in healthy people.^5^, ^6^ Nevertheless, many types of antibodies are found for which the meaning remains unclear and controversial.^4^, ^6^ We focus in this review on antibodies directed against dense fine speckled 70 protein (anti‐DFS70), known for several years but now detectable by a number of laboratories performing commercial testing, reviewing their clinical‐serological associations in CTDs.

## 2 ANTINUCLEAR ANTIBODIES

The importance of ANAs and their interpretation depends on both the type of antibodies and the autoimmune disease in which they occur.^5^ A negative ANA antibody result practically excludes an autoimmune disease. Although various CTDs are separate clinical entities, they may share some clinical features. Therefore, in each CTD there are specific autoantibodies, as well as antibodies also appearing in other diseases. Sometimes patients present elements of the clinical picture common to several different CTDs.^8^

Some ANAs, due to their high specificity toward certain disease entities, are universally used as their diagnostic markers, specifically antibodies directed against double‐stranded DNA (anti‐dsDNA) in systemic lupus erythematosus (SLE).^5^, ^8^ It was shown that ANA‐positive individuals had higher pro‐inflammatory cytokine levels in their serum, such as interleukin‐6 (IL‐6) and interleukin‐8 (IL‐8), compared with ANA‐negative individuals. Thus, it can be assumed that elevated ANA titers have an effect on immune system dysfunction, which in turn promotes the progression of CTDs. Despite this, the majority of ANA‐positive patients will not develop full‐blown disease.^5^

The determination of ANAs in the diagnosis of CTDs will bring the greatest benefits while considering the clinical picture of the patient and the association of antibodies with different diseases.^9^ However, in a majority of cases titer does not correlate with a disease flare. Therefore, multiple testing for ANAs to monitor the course of CTD is of little value.^8^

## 3 ANTI‐DFS70 ANTIBODIES

**Figure 1:**
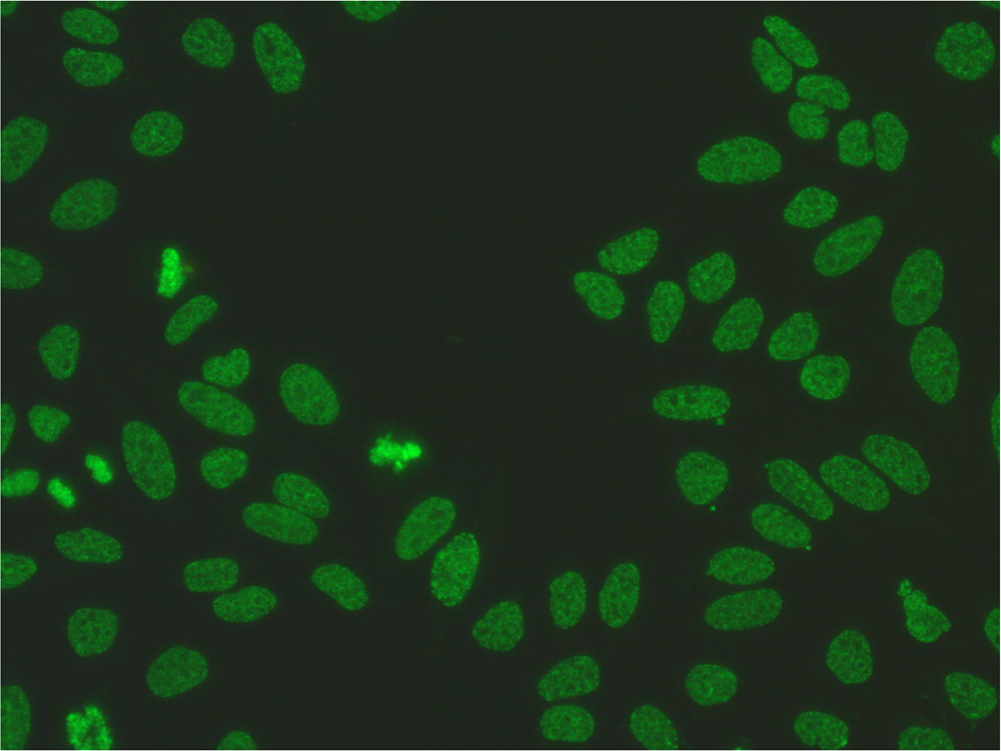
DSF70 Speckled pattern distributed throughout the interphase nucleus with characteristic heterogeneity in the size, brightness, and distribution of the speckles. Note some denser and looser areas of speckles (courtesy of Marzena Kraska‐Gacka).

The ANA subtype anti‐DFS70 occurs mainly in the class of immunoglobulins type G (IgG), whose name derives from the image of dense, fine, speckled fluorescence in IIF (Figure 1). DFS70 protein with a molecular weight of 70 kDa is widely expressed in many different tissues. This antigen has been identified as lens epithelium‐derived growth factor.^4^

Anti‐DFS70 antibodies were first described in association with interstitial cystitis. The DFS pattern in IIF is also observed in diseases such as atopic dermatitis, psoriasis, alopecia areata, prostate cancer, and asthma. This protein may participate in cell protection against oxidative DNA damage due to the influence of various environmental factors, thereby promoting cell survival.^10^

The correct interpretation of the DFS pattern in IIF requires considerable precision and experience due to the difficulties in differentiating it from other patterns, especially a homogeneous nuclear pattern.^11^, ^12^

Current data linked with clinical‐serological correlations revealed some controversial findings.^3^ Some authors claim that patients with CTDs are characterized by a low incidence of the DFS pattern observed in IIF with circulating anti‐DFS70 antibodies, while others state that the presence of these antibodies is not a sufficently reliable factor to clearly exclude a CTD.^11^ Still other authors state that anti‐DFS70 autoantibodies are present at a very low frequency in CTDs compared with other inflammatory diseases or healthy populations. But to date, it is unclear as to what extend the presence of anti‐DFS70 can reliably rule out CTDs.^10^, ^11^, ^12^ Moreover, some researchers postulated that these antibodies are detected more frequently at higher titers and without concomitant antibodies directed against extractable nuclear antigen (anti‐ENA) in healthy subjects (HS) compared with patients with CTD.^13^ It is also uncertain how to interpret the risk of CTD diagnosis in correlation with the titer of anti‐DFS70.^12^ However, the existence of pseudo‐DFS autoantibodies suggests the value of confirmatory testing.^2^

## 4 SLE

SLE is an autoimmune‐mediated CTD manifested by a wide range of clinical signs from various organs and also by the presence of serum ANAs, which account for the entry criterion to classify SLE without any scoring value according to the 2019 ACR/EULAR classification criteria.^14^, ^15^ Due to the diverse course of the disease among ANA‐positive patients, there is a high risk of delaying an accurate diagnosis because of the clinical similarity of many diseases to SLE.^14^

Aragon et al.^16^ in their prospective study showed that isolated anti‐DFS70 are present in significantly higher concentrations in HS (21.33%) than in SLE patients (3.1%). Therefore, the authors postulate that monospecific anti‐DFS70 antibodies may be useful in the exclusion of the diagnosis of SLE in ANA‐positive patients with not classified arthritis, muscle pain, or unspecific skin lesions.^14^, ^16^ The high level of these antibodies in a healthy population suggests that, if they are present in SLE patients, they can have a protective function and promote an enhanced response to SLE treatment.^17^, ^18^

Dai et al.^19^ evinced that the presence of anti‐DFS70 autoantibodies in SLE patients is positively correlated with younger age, higher anti‐dsDNA level, reduced C3 and C4 concentration, an elevated erythrocyte sedimentation rate (ESR), mucosal ulcers and leukopenia. Those laboratory parameters are considered to be the indicators of the active phase of SLE. These results suggest also a positive association between the presence of anti‐DFS70 and anti‐dsDNA.^19^

Different types of ANAs are associated with particular clinical manifestations of lupus. The level of anti‐dsDNA antibodies changes with progression and correlates with the severity of lupus nephropathy.^20^ The protective role of anti‐DFS70 antibodies toward kidney involvement is controversial.^21^ Interestingly, Chen et al.^21^ showed that patients with lupus nephritis (LN) and were positive for anti‐DFS70 antibodies required angiotensin converting enzyme inhibitors and aldosterone antagonists less frequently than LN patients without these antibodies, which implies a protective function toward the kidney. Concurrently, there was an increased incidence of proliferative lupus nephritis (PLN) in LN patients with anti‐DFS70 antibodies, which were associated with higher anti‐dsDNA antibody levels and impaired renal function, contradicting the previous hypothesis of protective anti‐DFS70 activity in LN patients.^21^

Moreover, studies show that the clinical picture of SLE patients with current anti‐DFS70 antibodies is characterized most by arthritis and photosensitivity, while the results of laboratory tests are more likely to document hemolytic anemia.^17^

Further long‐term studies may be crucial for understanding the diagnostic and prognostic significance of anti‐DFS70 antibodies in SLE.

## 5 SJÖGREN'S SYNDROME (SS)

SS is an autoimmune disease in which lymphocytic infiltration of the salivary and lacrimal glands leads to mouth and eye dryness.^22^ These symptoms are nonspecific and may also occur during infection, in the elderly and as a side effect of pharmacological therapy, which makes the diagnosis difficult.^14^

A study conducted by Hayashi et al.^23^ in a Japanese cohort provided information on the prevalence of anti‐DFS70 antibodies in patients with serum ANA‐associated rheumatic diseases (AARD). The results indicated that SS is the second to SLE as the disorder with the highest incidence of anti‐DFS70 (21.3% vs. 22.1%).^23^

Conticini et al.^24^ retrospectively assessed the frequency of anti‐DFS70 antibodies in a cohort of patients who underwent minor salivary gland biopsy and ANA testing to evaluate possible primary Sjögren's syndrome (pSS). Patients affected by other CTDs and infectious diseases were excluded from the study. In this analysis, anti‐DFS70 frequency was 1.82% among ANA‐positive subjects, 1.08% among pSS patients, and 0.45% among HS. Conversely, seven out of nine (77.8%) anti‐DFS70 positive individuals received a diagnosis of pSS, while only two of nine (22.2%) had no definite diagnosis established. We concur with the authors' conclusion that laboratory results should be interpreted in the context of clinical findings. However, as there was a relatively small sample size, based on this study alone, the presence of anti‐DFS70 antibodies should not be considered as an exclusion criterion neither for CTDs nor pSS.^17^, ^24^, ^25^

## 6 SYSTEMIC SCLEROSIS

SSc is a CTD with a complex etiology characterized by fibrosis of the skin and internal organs. The key factors in its pathogenesis are blood vessel and immunological abnormalities and tissue sclerotization.^26^

Numerous studies have shown that ANAs occur with a frequency of over 95% among SSc patients. Considering the relationship between ANAs typical for SSc, such as antibodies directed against topoisomerase I (anti‐Scl‐70) with the clinical picture of the disease, it seems reasonable to determine the specificity of ANAs in the routine diagnosis of SSc, especially in the early stage of the disease.^14^

Individuals with SSc rarely exhibit anti‐DFS70 antibodies (1.5%) which were also observed to occur only when SSc‐specific autoantibodies tested positive. Thus, in anti‐DFS70 positive patients with negative SSc‐specific ANAs, the diagnosis of SSc can be ruled out with a reasonable probability.^14^

Pulmonary complications such as interstitial lung disease (ILD) are relatively common during SSc.^27^ A study conducted by Lyu et al.^27^ observed that anti‐DFS70 antibodies were identified more often in HS compared with ILD patients. ANA‐positive and anti‐DFS70‐negative patients with ILD showed more frequent progression of symptoms to any specific CTD compared with those with confirmed presence of anti‐DFS70 antibodies. In addition, in the group of ILD patients with positive ANAs, the prevalence of anti‐DFS70 was higher in patients with idiopathic interstitial pneumonia (22%) than in those with follow‐up CTD (16%). Consequently, we can assume that the risk of ILD associated with the subsequent development of SSc is lower in the case of positive anti‐DFS70 antibodies.^27^

Although ANAs are an important diagnostic marker in SSc, the available literature provides little information about the impact of anti‐DFS70 antibodies on the course of SSc.^28^ Therefore, it is not possible to unequivocally determine whether isolated anti‐DFS70 in the group of ANA‐positive individuals excludes the diagnosis of SSc. Accordingly, new research projects may be needed to clarify this matter.

## 7 IDIOPATHIC INFLAMMATORY MYOPATHIES (IIM)

IIMs are a broad group of autoimmune diseases including dermatomyositis (DM) and polymyositis (PM). A representative feature of IIM is proximal muscle weakness and elevated serum creatine kinase levels.^29^ The classification of IIM subtypes is largely based on the presence of myositis‐specific antibodies (MSA).^14^

A number of works have evaluated anti‐DFS70 antibodies in IIM. The study by Hayashi et al.^23^ suggests that the incidence of anti‐DFS70 antibodies is lowest in DM/PM patients compared with other AARDs and is 3%, whereas in SLE, mixed connective tissue disease (MCTD), SSc, SS, and rheumatoid arthritis (RA), it was respectively 22.1%, 14.3%, 14.3%, 21.3%, and 18.1%. Mercado et al.^30^ included 71 patients with DM, 47 patients with RA, 30 obese patients, and 23 HS in addition to the rare presence of anti‐DFS70 in the DM group (1.4%), which was compared with HS (17.4%). They found that the most commonly observed ANA antibody pattern in IIF was nuclear fine speckled (NFS) imaging, which should be differentiated from the DFS‐IIF pattern. As many as 31 patients with DM (43.7%) presented the NFS pattern.

Muro et al.^31^ examined blood serum samples from 116 DM patients in a Japanese population. The following subtypes of DM were distinguished in the study group: clinically amyopathic DM, cancer‐related DM, classic adult DM, and juvenile DM. Five out of seven anti‐DFS70 positive patients with DM were also positive for MSA, strongly suggesting that the relatively rare presence of anti‐DFS70 antibodies in DM is positively correlated with the presence of MSA, for example, anti‐melanoma differentiation‐associated gene 5 antibodies (anti‐MDA5). These antibodies are most typical for clinically amyopathic DM with concomitant ILD. Among four DM patients with anti‐DFS70 and anti‐MDA5 antibodies, three patients survived and one patient died due to the ILD progression. In the deceased patient, anti‐MDA5 antibody levels were stable, but anti‐DFS70 antibody titers were significantly reduced. In the remaining three alive patients, the level of anti‐MDA5 became undetectable and the level of anti‐DFS70 increased, which followed clinical remission of ILD or amyopathic DM. These results imply that anti‐DFS70 antibodies may inhibit the progression of DM.^31^

The involvement of anti‐DFS70 antibodies in the process of excluding suspicious cases of DM/PM as well as in alleviating the clinical symptoms of IIM is probable but needs to be confirmed in further long‐term prospective studies.

## 8 UNDIFFERENTIATED CONNECTIVE TISSUE DISEASE (UCTD)

UCTD is an autoimmune disorder that does not fulfill the classification criteria necessary for diagnosis of a specific CTD.^32^ UCTD is most commonly manifested by pain and inflammation of the joints.^33^ The prevalence of anti‐DFS70 antibodies among patients with UCTD is estimated at 8%–40% and they usually coexist with antibodies typical for specific CTDs.^34^

It has been proven that 1/3 of patients with UCTD will develop fully symptomatic CTD within 5 years from the onset of the first manifestations. Therefore the search for serological markers that will help exclude the diagnosis of UCTD with a high risk of progression to CTD has been undertaken. Anti‐DFS70 antibodies are thought to have this function.^34^ Infantino et al.^33^ evaluated 91 patients diagnosed with UCTD, documenting a high prevalence of anti‐DFS70 antibodies in this study group (13.3%). Given that the presence of these antibodies is not typical for CTDs, anti‐DFS70 appears to be a reliable marker for UCTD and indicates a low risk of specific CTD developing.

Based on available economic analyses, it can be concluded that the use of anti‐DFS70 antibody assay in the standard management of patients with suspected UCTD is economically beneficial, as it reduces expenses related to unnecessary laboratory and imaging diagnostics.^35^

Controversial clinical data do not fully support the validity of anti‐DFS70 assay for the diagnosis of UCTD. The coexistence of other autoantibodies may suggest a risk of developing CTD in the future.

## 9 CONCLUSIONS

Previous studies indicate that anti‐DFS70 antibodies occurring in the blood serum in high concentrations, and as the only ones, are a reliable marker for excluding CTDs. They constitute a differentiating factor between healthy people and CTD patients among ANA‐positive patients. Accordingly, anti‐DFS70 screening tests should be included in ANA detection algorithms.^13^ Such a procedure will make it possible to reduce unnecessary expenses for further needless examinations and will also improve the diagnostic and therapeutic process.^36^ However, the DFS pattern is generated by various ANAs. Therefore, the IIF technique detecting anti‐DFS70 is burdened with a significant number of false positive results. For correct interpretation of test results, anti‐DFS70 assays with higher specificity, for example, chemiluminescence immunoassay (CLIA), enzyme‐linked immunosorbent assay (ELISA), fluoroenzymatic immunoassay (FEIA), immunoblot or modified IIF assays, should be taken into consideration.^6^, ^37^, ^38^, ^39^

## AUTHOR CONTRIBUTIONS

**Aleksandra Fijałkowska**: Conceptualization; investigation; methodology; writing—original draft; writing—review and editing. **Robert A. Schwartz**: Conceptualization; project administration; supervision; writing—original draft; writing—review and editing. **Anna Woźniacka**: Conceptualization; methodology; project administration; supervision; writing—original draft; writing—review and editing.

## CONFLICT OF INTEREST STATEMENT

The authors declare no conflict of interest.

## Data Availability

The data that support the findings of this study are available from the corresponding author upon reasonable request.
